# Preparing for Pediatrics: Experiential Learning Helps Medical Students Prepare for Their Clinical Placement

**DOI:** 10.3389/fped.2022.834825

**Published:** 2022-03-04

**Authors:** Clare Sullivan, Claire Condron, Claire Mulhall, Mohammad Almulla, Maria Kelly, Daire O'Leary, Walter Eppich

**Affiliations:** ^1^RCSI SIM Centre for Simulation Education and Research, RCSI University of Medicine and Health Sciences, Dublin, Ireland; ^2^RCSI School of Medicine, RCSI University of Medicine and Health Sciences, Dublin, Ireland; ^3^REACH RCSI, RCSI University of Medicine and Health Sciences, Dublin, Ireland; ^4^Department of Pediatrics, RCSI University of Medicine and Health Sciences, Dublin, Ireland

**Keywords:** pediatrics, communication, simulation, experiential learning, simulated patient

## Abstract

Despite the importance of effective communication skills in pediatrics, clinical placements may inadequately prepare undergraduate students to communicate with children. The integration of non-clinical interactions with healthy children within a pediatric curriculum has the potential to enhance learning. We designed and implemented a novel course involving experiential learning, including video-recorded consultations with simulated parents (SPs), team-based scenarios with a pediatric mannequin, interactions with healthy children through a pre-school visit and medical student led health workshops for primary school children. Medical students at the RCSI University of Medicine and Health Sciences took part in the course. We used a mixed methods approach to assess the impact of the course. We investigated medical students' perspectives through a pre- and post-intervention questionnaire and post-intervention focus group discussions (FGDs). We assessed participating children's health literacy at the start of the course. 144/279 (51.6%) of the fourth year medical student cohort on their pediatric rotation, consented to participate in the study. All 144 (100%) of consenting students completed the pre-intervention questionnaire. 59/144 (40.1%) of consenting students completed the post-intervention questionnaire. Results showed a statistically significant improvement in ratings (*p* < 0.05) for items related to managing a confrontational situation involving family members, completing a psychosocial assessment with an adolescent and effectiveness using evidence-based medicine (EBM) when motivating patients. There was a statistically significant decrease in how students rated their comfort at using EBM when motivating patients. Four themes relating to how students experienced the intervention were identified from eight FGDs (*n* = 35 students): Shaping Student Learning; Supporting Student Learning; Developing New Skills and Feeling More Prepared. 39/49 (79.6%) children completed a health literacy assessment. All questions had a high percentage of positive responses. Question 7, understanding your doctor, had the highest proportion of negative responses (27%). Ours is one of the first studies to design an educational intervention to enhance pediatrics teaching by combining interactions with healthy children outside of a clinical setting with more traditional simulation-based approaches. We conclude that this type of intervention supports students' learning of pediatric communication skills and enhances students' perceived preparation for clinical placement.

## Introduction

Despite the importance of effective communication in pediatrics (1), clinical placements may inadequately equip undergraduate medical students with the opportunity to learn effective pediatric communication skills (2). During clinical placements, interactions with children are often observational (3), meaning that most students have only limited interactions with children unless they are involved with children in external activities or through families and friends. While medical students are often eager for real patient contact, when presented with such opportunities through clinical placements they can find the transition abrupt (4). The start to clinical placements can be unpleasant for students due to their lack of knowledge related to specific specialties, their role and the new environment (5). Furthermore, medical students experience additional struggles related to frequent changes in staff and difficulties applying knowledge in practice (6). Some of these challenges are common across all clinical placements, others are unique to a specific specialty (7). Those unique to pediatrics relate to the significant variability in how consultation skills are taught during pediatric clerkships (8) and the aspects of pediatric communication skills which add additional complexity such as the triadic nature of the consultation between the child, the parent or caregiver and the doctor and the different communication strategies required for children of different developmental stages (9). While observational learning can be effective (10), students must draw on certain attributes such as self-regulation, self-efficacy and insight. If students lack these skills and do not have the required knowledge to make sense of this observational learning, are students potentially missing out on rich learning opportunities?

Communication skills training has the potential to bring significant benefits not only to participants but also to their patients and families (3). To be effective, students must develop the unique skills to communicate with children and have opportunities to practice in settings that do not interfere with patient care (2). Experiential learning represents an ideal setting for communication skills training (11). The main theoretical underpinning for experiential learning is Kolb's Experiential Learning Cycle (12). This model includes four stages: (a) concrete experience: actively participating in an experience; (b) reflective observation: reflecting on the experience; (c) abstract conceptualization: translating the reflections to learnings; and (d) active experimentation, trying out what has been learned (12). This theory focuses on learning from one's own concrete experience and does not address learning that occurs through observation of other learners' experiences. More recent theories highlight the value of vicarious experiential learning, or learning through the observation of others' in action (14). In simulation-based learning, when learners are observing peers, their observations should be structured through the use of tools to optimize learning (15), perhaps a feature of observational learning which could also be transferred to observations in the clinical setting. Therefore in experiential learning settings, learning is most effective when learners both participate themselves and engage in structured observation of their peers.

While pediatric clerkship directors believe simulation-based education meets teaching requirements, they also have a number of concerns, including: (a) barriers to its implementation such as funding, available faculty time, technical support, lack of simulation trained faculty and availability of physical space, and (b) appropriateness of replacing real experiences with simulated experiences (16). While experiential learning in healthcare education often focuses either on simulation-based or workplace-based learning, we see additional scope to enhance experiential learning with the inclusion of real interactions outside clinical settings. Challenges exist however concerning the involvement of children as laws prohibit the employment of children in the same format as adult simulated patients. While medical programmes have successfully integrated children as patients into pediatric teaching and assessment and shown positive outcomes for both the children and learners, ethical concerns remain (17). Recommendations suggest limiting this type of involvement to assessments that cannot be carried out by other means (18). Partnerships between a school and a university can provide a solution in the context of pediatric skills (19) where an intervention is designed in such a way that it is mutually beneficial to both students and children.

We hypothesized that providing experiential learning experiences for students outside the clinical setting, through simulation and community engaged learning has the potential to better prepare undergraduate medical students for their pediatric clinical placements. To this end, we designed and implemented a novel course that integrated simulation-based learning and interactions with healthy children through student led health and well-being workshops. Our aims were to explore the impact of these learning experiences on medical students' development of pediatric communication skills and their preparedness for clinical placement as well as the impact of the workshops on the children's health literacy.

## Methods

### Development of the Novel Pre-clinical Course

The experiential learning week was held in the RCSI SIM Centre for Simulation Education and Research. The teaching had taken place for the first time in the 2018/2019 academic year, and the study took place during the second year of the teaching in the 2019/2020 academic year. The course represented the refinement of an existing curriculum based on feedback from previous students and an assessment of knowledge gaps identified during previous clinical assessments. Common deficits identified within the communication portion of the clinical examination informed this educational innovation for teaching communication skills. We aimed to minimize didactic teaching and emphasize active student engagement.

The 5-day course was designed to be placed at the start of the 7-week pediatric clinical rotation. See Table 1 for an overview of the experiential learning elements and respective learning outcomes. The aim was to give students experience with the basic skills they would need for placement and to ensure exposure to all the required elements even if experiences on placement differed. The week involved multiple teaching modalities, however, it was the four experiential learning elements which were the focus of this study. These were recorded simulated parent (SP) consultations, medical student led health and well-being workshops, team-based scenarios with a pediatric mannequin and a pre-school visit (see Table 1). The main aim was to foster appropriate communication skills for varying contexts including other healthcare team members, children and their families and carers. The students participated in the SP encounters on day 2, the health and well-being workshops on day 3 and team-based simulations on day 4. As the pre-school could only accommodate a small number of students at a given time, students attended the pre-school on day 2 or day 4 depending on the time they were allocated.

**Table 1:** Course elements and learning outcomes.

**Course Element**	**Learning Outcomes**
Recorded simulated parent consultations (without a child present) with multi-source feedback (self, tutor and simulated parent)	Demonstrates the ability to accomplish the specific tasks of an effective consultation. (i) Establishes and builds a relationship (ii) Initiates the consultation and sets the agenda (iii) Establishes, recognises, and meets patient needs (iv) Gathers information (v) Explains the diagnosis and plans and negotiates management plans (vi) Structures, and prioritizes the consultation (vii) Closes the consultation and establishes future plan
A health and well-being workshop delivered by the medical students to local primary school children (aged 7–9 years)	Demonstrates the ability to convey specific explanations related to health promotion and EBM in an age appropriate manner Establishes and builds a relationship
Team based simulated scenario with a pediatric mannequin, which was live streamed for peer learning	Demonstrates the ability to take a systematic, problem-focused medical and surgical history and interpret the relevant clinical findings Interprets and integrates the history, findings of physical examination, results of laboratory tests and imaging studies, and other relevant data to arrive at an appropriate diagnosis or differential diagnosis Demonstrates effective communication skills with members of the health care team
A pre-school visit to observe children at different levels of development (aged 6 months to 4 years)	Demonstrates age appropriate communication skills for children of different developmental stages Identifies the normal developmental milestones and differences in children's stages of development

Experienced pediatric faculty had written the SP cases and the team-based simulation scenarios the previous year and the same scenarios were used in the teaching. See Appendix A for details of the cases and scenarios. All SPs who participated in the teaching had completed RCSI simulated patient training (Appendix B). Their case was emailed to them 4 days before the teaching. They were given the opportunity to clarify details on the day. Each scenario lasted 8 min and was followed directly by 2 min of verbal feedback from the SP to the student. The scenarios were video recorded using a web-based audio-visual recording and learning platform (CAE Learning Space, Sarasota, Florida). Immediately after the consultation and verbal feedback, the SPs entered feedback on the learning platform using the Consultation and Relational Empathy (CARE) measure (20). Students were in groups of three or four. They participated in one consultation individually and then watched their peers. Afterwards students watched back their own video and rated themselves on the learning platform using a subset of questions from the Pediatric Consultation Skills Assessment Tool (PCAT) (1) (Appendix C). Faculty also rated the students' videos with the PCAT. Students were given access to the faculty and SP ratings through the learning platform on completion of their self-rating.

Since teaching took place outside the clinical setting, it was important to integrate direct experience with children. Given ethical challenges with the involvement of children as simulated patients, we explored other means of providing opportunities for medical students to engage with children. We created opportunities in two ways across two different age groups. By partnering with two local schools, we created a community engaged learning experience (21) which was mutually beneficial to both medical students and children. We organized medical student led health and well-being workshops for primary school children (aged 7–9 years). The schools proposed health-related topics that would be relevant to the children. Through the workshop delivery, medical students had the opportunity to learn how to explain health-related topics at the appropriate development stage for children, in language they could understand. The children had both the opportunity to learn about health and well-being and the opportunity to attend third level campus regularly. Familiarization with a third level campus improves access to higher education by breaking down barriers and allowing the children to interact with university students and staff (21). The day before the workshop, students were briefed on their role, divided into groups, given guidelines regarding the format and topic of the workshop and provided with a lesson plan template. Medical students were encouraged to make the workshops interactive and choose from arts and crafts, equipment and technology to support the workshop. The following day, in advance of the workshop, faculty reviewed the appropriateness of students' plans. The children attended the RCSI SIM Center for Simulation Education and Research with their teachers and were divided into small groups of four to six with the medical students (ratio of 1:2 or 1:1 medical students to children). Teachers, community engagement staff, pediatric faculty and simulation staff were present at all times but gave minimal input to the workshops once started. The 90 min workshop included a fruit break after 45 min.

During the team based simulations, students worked in groups of three to manage an acutely unwell child. One faculty member controlled the parameters of the pediatric mannequin (Gaumard Super Tory Mannequin) in a separate control room and acted as an embedded participant for telephone calls. A second faculty member acted as an embedded participant in the role of a nurse within the treatment room. The session was live streamed to students watching in another room. Students participated in one scenario and watched three others. Each scenario lasted ~15 min and was followed by a 30 min group debrief with faculty immediately after. Debriefings included feedback on team communication and decision-making and addressed students' specific questions about medical care.

To give students a chance to interact with younger children and to understand the stages of development, we arranged a visit for the students to a local pre-school (aged 6 months−4 years). During this visit, we provided students with a worksheet of prompts regarding the different domains of development to help structure their observation of the children. We also encouraged them to discuss their observations with their peers.

The teaching week was repeated five times throughout the year to cover all five rotational blocks of students. Each block was further subdivided into two groups, so the teaching was repeated twice in each block. The fifth rotation was excluded from analysis since it had significant modifications due to restrictions on educational activities as a result of the COVID-19 pandemic.

### Evaluation of the Impact of the Course

This cross-sectional mixed methods study, explored the learning experience of students participating in pediatric teaching during the 2019/2020 academic year.

We conducted a mixed methods analysis of the medical students' perspectives of pre-clinical experiential learning using focus group discussions (FGDs) and a questionnaire administered before and after the intervention. A mixed methods approach is particularly useful when studying complex initiatives and can also allow for the better application of findings (22). Kolb's experiential learning theory (12), as well as theories on vicarious experiential learning (14) were used as sensitizing concepts in the analysis due to their relevance in explaining the learning processes in experiential learning settings. Our aim with the FGDs was to understand how students learned from the experiential learning elements of the course and in what ways the course impacted their perspectives toward their pediatric clinical placement. The pre-post questionnaire was self-assessed to help us understand the change in students' perceived abilities as a result of the course. Better understanding of what aspects of the intervention students found effective for learning would allow us to further adapt and improve the teaching.

We also assessed participating children's health literacy at the start of the course. We planned to complete the health literacy assessment at the end of the course but the immediate shut down of education at the start of the COVID-19 pandemic meant this was not possible. The study was approved by the Royal College of Surgeons in Ireland (RCSI) research ethics committee REC001719.

### Participants

Study participants were undertaking their pediatric clerkship during their penultimate year in the medical school at RCSI. All medicine programmes (undergraduate 5 and 6 year, graduate entry 4 year) are together at this point. All medical students attending the pediatric rotation were eligible to participate in the research and all students received the teaching intervention regardless of participation in the research. Convenience sampling was used.

Through the Recreation Education And Community Health (REACH) RCSI Programme, the university community engagement and access programme, children from two local schools were invited to participate in the workshops. The schools were DEIS schools (Delivering Equality of Opportunity in Schools), identified as educationally disadvantaged by the Department of Education in Ireland. Children were in third class of primary school. All children in the invited classes participated in the workshops regardless of participation in the research.

### Data Collection

**Figure 1:**
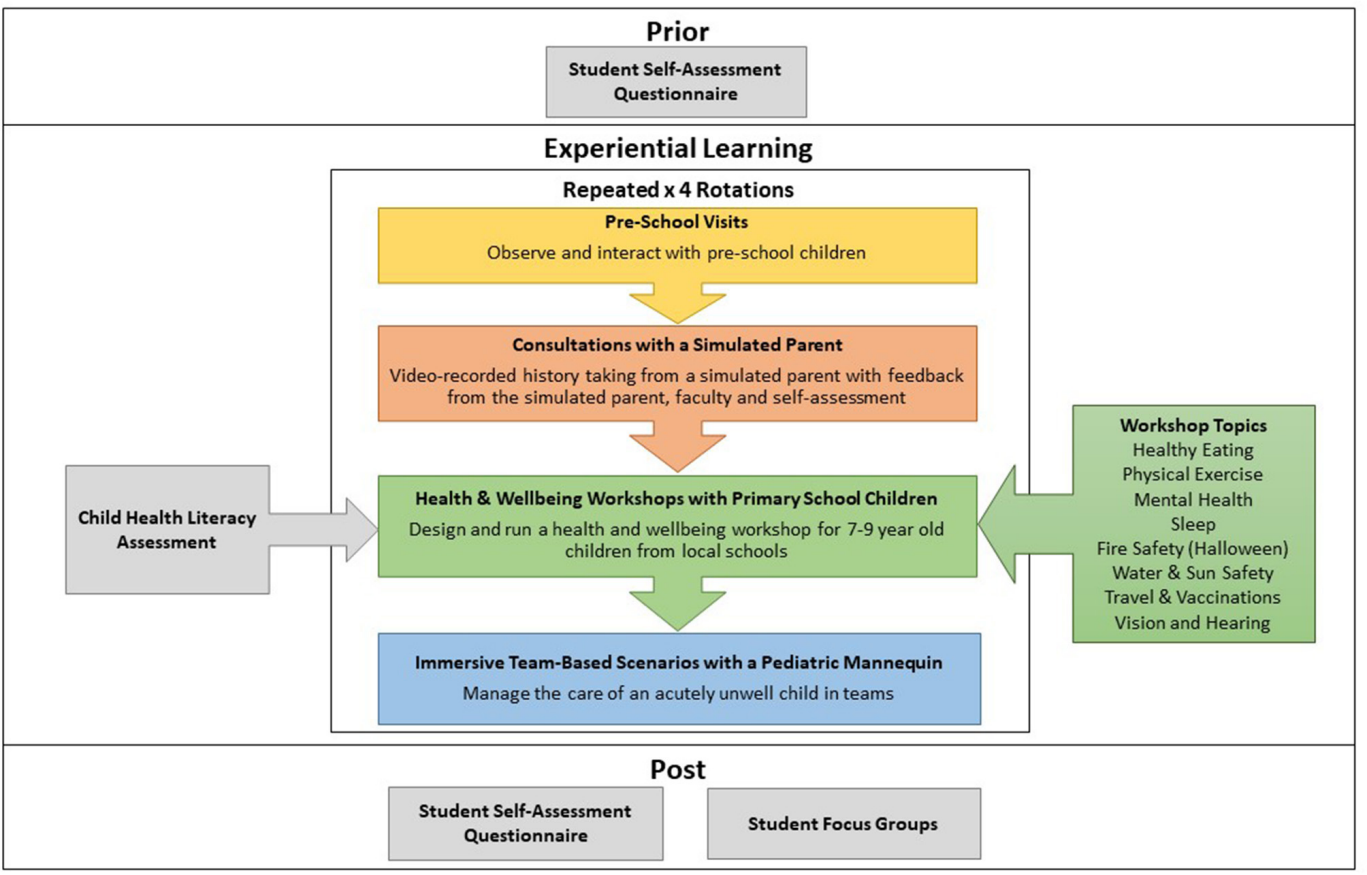
Overview of intervention and data collection.

We collected various forms of quantitative and qualitative data (see Figure 1). Before and after the intervention students completed a questionnaire about their perceived ability/knowledge in certain areas of pediatrics (23) (Appendix D). We held FGDs with students at the end of each teaching week. FGDs were selected for the collection of qualitative data as these have become a method of choice for assessing programmes (24). The FGDs were between 30 and 45 min long and were conducted by the lead researcher (CS) who supported the programme but was not in involved in grading the students. Another researcher (CM) was also present who managed consent and took field notes. See Appendix E for the interview topic guide. The FGDs were audio recorded, transcribed and de-identified.

**Figure 2:**
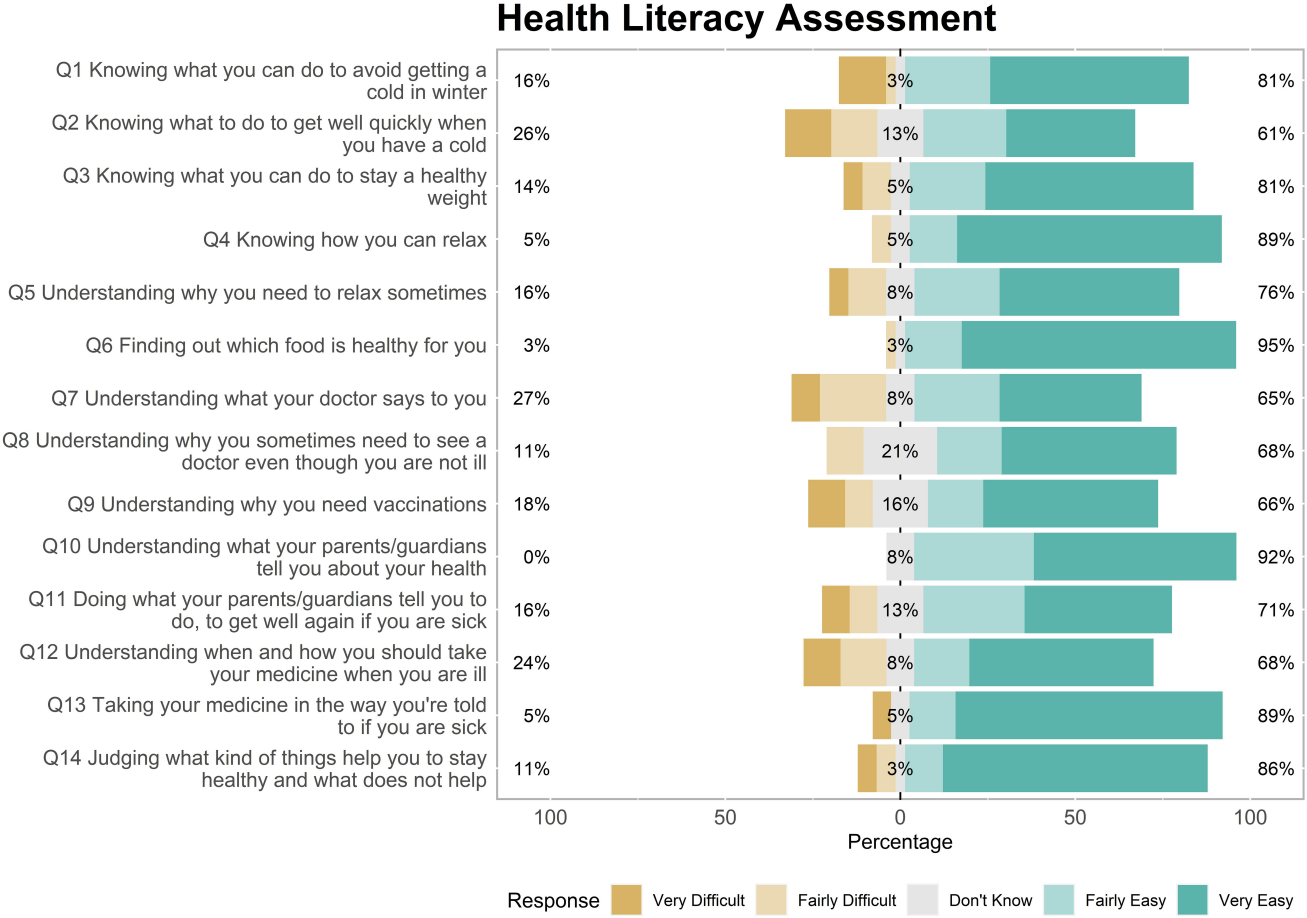
Responses to health literacy assessment.

Children who participated in the health and well-being workshops and who gave consent (both parent/guardian consent and child assent) were asked to complete a health literacy assessment at the start of the intervention. The health literacy assessment was based on the European Health Literacy Survey adapted for this age-group (25). While the tool had been validated only in the German language, an English translation was available and was used due to the lack of other suitable tools available at the time. The language in the English translation was reviewed by the research team, including the lead researcher (CS), the community engagement manager (MK) and a pediatric tutor (DOL). The school teachers were also asked if they thought the language was suitable for their class groups. Finally, the instrument was piloted with a small number of children not involved in the study to test for readability in the relevant age group. Following this process, a revised version of the tool was created and used for data collection (Figure 2). The main changes included; re-ordering the questions so that related items were beside each other; changing the format of the question from “How easy or difficult is it for you to …” to “Do you find each of the following easy or difficult?” and removing the question “stick to what you have learned in road safety lessons or the safe cross code?”, as this was not directly related to health.

### Data Analysis

#### Quantitative Data Analysis

Statistical analysis was conducted using the statistical package R (R Core Team, 2019, Vienna, Austria). Pre and post intervention questionnaire responses were compared using the paired sample Wilcoxon signed rank test with Bonferroni adjustment (26). Descriptive statistics were used to summarize the children's responses to the health literacy assessment completed at the start of the intervention.

#### Qualitative Data Analysis

Data were analyzed using thematic analysis (27). Data were coded inductively using the theories of experiential and vicarious learning as sensitizing concepts. Preliminary data coding was performed by two researchers (CS and MA). CS was experienced in coordinating simulation-based education (SBE) and MA was an undergraduate medical student who had not participated in the intervention. The addition of a medical student to the research team aimed to include the student perspective. After initial familiarization with the data, first-level coding was completed independently by two of the researchers on the first two FGDs. CS completed analysis using NVivo (QSR International Pty Ltd., 2018, Version 12) and MA used Microsoft Word and Excel. After the preliminary coding, CS and MA met with two experienced simulation educators (CC and WE), one with significant expertise in qualitative research (WE), to review the analysis and refine the codebook through discussion to achieve consensus. The two researchers then coded the remaining six FGDs using the codebook before meeting again to discuss any new codes and to carry out second-level coding by agreeing on the grouping of codes into themes. The researchers ensured reflexivity by reflecting on the influence of their knowledge and experience on the analysis. Of note, in addition to expertise in simulation and qualitative research, WE is an experienced pediatric emergency medicine physician. The research team enhanced the credibility of the findings by ensuring their relevance and applicability to the teaching and by extracting quotes to illustrate main themes (28).

## Results

### Pre–post Questionnaire

Two hundred and seventy nine medical students across the four included rotation blocks completed the full course as it was a mandatory part of their pediatrics teaching. Of the 279 students, 144/279 (51.6%) of them consented to participate in the study. The 144 consenting students were across all four included rotations: 47 from the first rotation, 34 from the second rotation, 45 the third rotation and 18 from the fourth rotation. Students who consented to participate received the pre-post questionnaire and were invited to a FGD. All 144 (100%) of consenting students completed the pre-intervention questionnaire. 59/144 (40.1%) students completed the post-intervention questionnaire. Of the 59 who complete both pre and post-intervention questionnaires, 10 students were from the first rotation, 14 from the second rotation, 22 from the third rotation and 13 from the fourth rotation.

The median and inter-quartile range for all except one question on the pre-intervention questionnaire were the same for the 144 cohort of students as for the subset of 59 students. Item seven was the only item which differed, having a median of 6 for the full group of 144 and a median of 5 for the subset of 59 students, however, the interquartile range was the same for both groups (4–7). As the responses of the subset of students were similar to that of the larger group, and to allow for pairwise comparison, further analysis was carried out only on the subset of 59 students who had completed both the pre and post questionnaires.

On the pre-intervention questionnaire the median student self-assessed rating was five or below (on the 10 point scale) for six of the eight items. The students rated themselves a six or higher on six of the eight items on the post-intervention questionnaire. Comparing the responses pre and post, after Bonferroni adjustment, items 4, 5, and 8 showed a statistically significant improvement in ratings (*p* < 0.05). These related to effectiveness at managing a confrontational situation involving family members (Q4, *p* = 0.01), comfort at completing a psychosocial assessment with an adolescent (Q5, *p* = 0.00003) and effectiveness using EBM when motivating patients (Q8, *p* = 0.0003). There was a statistically significant decrease in how students rated their comfort using EBM when motivating patients (Q7, *p* = 0.003). While not statistically significant, students rated themselves higher at the end of the week than at the start of the week on the four other questions (Table 2). Looking at the change in ratings before and after, for the overall group, the median change was an improvement for six of the eight questions, no change for the question concerning effectiveness at completing a psychosocial assessment with an adolescent (Q6) and a decrease in score for the question concerning comfort at using EBM when motivating patients (Q7). This pattern seems to follow across rotations two, three and four except for two differences: the median change was a decrease for the question concerning comfort communicating with parents and families (Q1) in rotation four; and there was an improvement for the question concerning effectiveness at completing psychosocial assessment with an adolescent (Q6) in both rotation two and rotation four. The results for rotation one appear different from the other rotations, reporting an improvement only on the question concerning comfort at completing a psychosocial assessment with an adolescent (Q5).

**Table 2:** Pre- and Post-intervention questionnaire results comparison.

	**Pre-intervention**	**Post-intervention**			**Overall Delta**	**Rt1 Delta**	**Rt2 Delta**	**Rt3 Delta**	**Rt4 Delta**
**Question**	**Median (IQR)** **(*****n*** **=** **59)**	**Median (IQR)** **(*****n*** **=** **59)**	***p*** **-Value**	***p*****-Value** **(adj)**	**Median** **(*****n*** **=** **59)**	**Median** **(*****n*** **=** **10)**	**Median** **(*****n*** **=** **14)**	**Median** **(*****n*** **=** **22)**	**Median** **(*****n*** **=** **13)**
1	6 (5–8)	7 (6–8)	0.04	0.34	1	0	1	1	−1
2	6 (5–7)	7 (6–7)	0.09	0.79	1	−0.5	1	1	1
3	5 (3–6)	6 (4–7)	0.007	0.06	1	0	1	1	2
4	5 (3–6)	6 (5–7)	0.001	0.01^*^	1	0	1	1	3
5	3 (1–5)	6 (4–7)	0.000004	0.00003^*^	2	2	2	2	3
6	3 (1–5)	4 (1–6)	0.10	0.83	0	0	1.5	0	1
7	5 (4–7)	3 (1–6)	0.0004	0.003^*^	−2	−1	−0.5	−2.5	−2
8	5 (4–6.5)	7 (6–8)	0.00004	0.0003^*^	1	0	1.5	2	1

*IQR = (1st, 3rd interquartile range)*.

*p-Value presented from the two sided Wilcoxon Sign Rank Test for paired data, pre and post intervention*.

*p-Value (adj) presented is the Bonferroni adjusted p-value for 8 tests*.

* *Significance values at the level of p < 0.05*.

*Delta represents the median of the difference in score between post and pre*.

*Note that the median of the differences (Overall Delta) is not the same as the difference of the medians (Post-intervention Median minus Pre-intervention Median) in skewed data*.

*Rt represents the rotation grouping*.

*Questions from Whitt et al. (23)*.

*Q1. Please rate your comfort level in communicating with parents and family members of young children*.

*Q2. Please rate how effective you are at communicating with parents and family members of young children*.

*Q3. Please rate your comfort level in managing a confrontational situation involving family members with differing opinions*.

*Q4. Please rate how effective you are at managing a confrontational situation involving family members with differing opinions*.

*Q5. Please rate your comfort level in completing a psychosocial (HEADSS) assessment with an adolescent*.

*Q6. Please rate how effective you are at completing a psychosocial (HEADSS) assessment with an adolescent*.

*Q7. Please rate how comfortable you are at using EBM when motivating patients*.

*Q8. Please rate how effective you are at using EBM when motivating patients*.

*Scale: Not comfortable / effective 1 2 3 4 5 6 7 8 9 10 Very comfortable / effective*.

### Focus Groups

We conducted eight FGDs with a total of 35 students. These were spread across the four included rotation blocks. There were participants from rotation one (12), rotation two (5), rotation three (11) and rotation four (7). Our intention was to capture the experiences of students who participated in each of the rotations which occurred at different times throughout the year. Later rotations would have already completed other clinical rotations in advance of pediatrics. There were male (15) and female participants (20) and participants came from both undergraduate (26) and graduate programmes (9). We identified four main themes that characterized how experiential learning supported and shaped the students' development of pediatric communication skills (Table 3).

**Table 3:** Focus group discussion themes.

**Theme**	**Sub-Themes**
Shaping student learning	Drawing on experience Desiring exposure Having concerns
Supporting student learning	Engaging in peer learning Engaging in self-reflection Receiving timely multisource feedback Putting theory into practice Having a supportive environment Having a realistic/authentic environment
Developing new skills	Being a team player Dealing with the unexpected Engaging with children Identifying normal range Interacting with parents Providing age appropriate explanations Learning how to apply knowledge
Feeling more prepared	Getting a good foundation Experiencing graded exposure Changing perspectives Addressing fears Building confidence Developing a pediatric mind-set

#### Theme 1: Shaping Student Learning

Students came into the teaching week looking to gain experience and address preconceived concerns they had about their ability in pediatric clinical settings. The students used evocative words to share how they felt about their pediatric rotation, such as:

- “*daunting” S09F, FG1.2*
- “*nervous” S34F, FG4.2*
- “*worried” S29F, FG4.1*
- “*terrified” S06F, FG1.2*
- “*fear” S02M, FG1.1*
- “*scariest thing” S08F, FG1.2*

Students highlighted the abruptness of the transition to clinical practice, describing the process akin to being “*thrown into the deep end” S22M, FG3.2*.

The nature of the students' concerns were quite diverse. Some students were concerned about encountering an acutely unwell child in the hospital and being out of their depth. Other students had concerns about managing difficult situations with parents who were very demanding. Students feared they would not be able capture children's attention to engage well with them.

“*I was honestly terrified. I am not really a kid person... I'm kinda glad that we have this week because it's nice to have… almost like a little tutorial...” S06F, FG1.2*

Students also brought with them experience from interactions with children outside of their studies through babysitting, sports and family and this experience gave some students confidence.

“*... I have a niece and a nephew… I spend a lot of time with them. I find it relatively easy. I enjoy my time with them, communicating with them. I find getting information from them rather easy” S27M, FG3.2*

However, even the students who were confident interacting with children in extra-curricular activities were less confident in the medical setting. They desired practice speaking to children about more serious topics and learning how to manage the needs of both parent and child. They acknowledged how there would be additional challenges in situations where a child was very sick or distressed.

“*I had prior experience with it because I taught swimming lessons for four years. … and then I was a camp counsellor, … but I imagine communicating with a very sick child who is more distressed will be different.” S18F, FG3.1*

#### Theme 2: Supporting Student Learning

Students reported elements within the teaching that helped them learn. Students valued learning from their peers. Some students did not feel comfortable going straight into the simulation so they appreciated the opportunity to watch others first. They also valued seeing how others approached a situation and having the chance to talk through what they found difficult. Watching their peers helped students see the standard to which they should aspire; they appreciated being able to “*model [themselves] after someone who is really good” (S31M, FG4.1)*. Some students would have liked a demonstration from a tutor in advance of the simulation or more time to watch the sample videos as they were not confident about how to approach the consultation. The knowledge that they were being watched motivated students to put in more effort to present themselves well.

“*It's good that you have a few of us in the same room as well. It gives you a bit of context and you can like learn from the people before you.” S17F, FG2.2*

Students appreciated prompt feedback from multiple sources, including personal reflection. They valued how detailed the feedback was from tutors and appreciated receiving it in a timely fashion. They benefited from separating the learning from the doing, describing their difficulty in remembering exactly what they had done afterwards. The video recordings were very useful for them to actually see their actions. Students who had a natural tendency to be critical of themselves identified that the video helped give them reassurance and have a more balanced view of their performance which in turn helped improve their confidence. Even students who described watching their own video as “*cringy” (S04F, FG1.1)* or “*awkward” (S16F, FG2.2)* acknowledged that they learned from it. They indicated how useful it was for identifying subconscious non-verbal behaviors about which they were not aware.

“*... I'm always really hard on myself… So having the history recorded… and then watching the video I was like, okay, that actually wasn't that bad... so it's kind of given me a lot more confidence...” S13F, FG2.1*

The team-based simulations were not recorded, however, students commented that they would have liked this to be recorded also so they could objectively see their actions during the debrief.

“*If the debrief we did afterwards included the video where it's showing you, here you did this, now you did that. That might be good because then you could actually see … Oh yeah, we're so busy.” S03M, FG1.1*

The structured and supportive environment allowed students to put theory into practice in a safe way. Students valued learning in an environment where they could make mistakes without negatively impacting on patient care or their grades.

“*... I think that was the best part because I think you could easily learn then without being like, this is gonna mess up my score or this has so much riding on it… ” S09F, FG1.2*

Students in early rotations requested additional guidance in advance of the SP interaction despite the availability of resources such as videos on an e-learning platform. Students in later rotations reported the opposite, that the level of information provided to them in advance made the interactions too easy.

Students also valued realism. Elements of teaching which improved the realism or authenticity of the situation helped them engage better with the learning. Elements which detracted from realism caused confusion for the students.

“*Whereas the simulated neonates that moved and groaned that was incredibly realistic and really helped you feel like you were actually part of a proper team...” S02M, FG1.1*

#### Theme 3: Developing New Skills

Students described how they developed skills, many of which related specifically to communicating in a pediatric environment: engaging children; using age-appropriate language; identifying the normal range and interacting with parents. Students saw the parallels between what they were doing and how they would navigate conversations in the clinical setting. Students spoke about having to be more creative in their conversations with children, teasing information out and trying to judge how much information they could understand. They spoke about learning how to build rapport and engage children through play and visual communication with pictures and drawings. They noted how each child was unique and that different personalities required different approaches.

“*... it was good when we were explaining to the children about healthy eating to put it into terms that they can understand...It takes more effort than it does explaining something to an adult...” S04F, FG1.1*

“*... we were given an opportunity to interact with kids of different age groups...so you kind of have an idea of what to expect with the different age groups…” S05F FG1.2*

When interacting with SPs students spoke about learning how to adapt their approach to a consultation. They noted differences in approach when talking to an adult patient about themselves compared to a situation where they were talking to a parent about their child. They learned about how parents might feel in such situations and how to reassure them about their concerns. Some students saw the positives, identifying that parents could help them identify problems with their child, acknowledging in adult consultations third parties can only rarely describe how the patient has been.

They developed broader skills such as how to apply knowledge in practice, how to deal with the unexpected and how to be a team player. Students often felt unprepared for the situations they were presented with during the week but despite this feeling, they managed to navigate the situation successfully. Students gained insight into the uncertainty of clinical practice; “*it's not an exact science” (S07M, FG1.2)* and that it was a matter of “*getting comfortable with feeling uncomfortable” (S06F, FG1.2)*. They learned about having different roles in a team and how to approach conversations with more senior team members.

“*… when you're going through the phone call [to senior staff], there's this list of things that you're trying to follow, but just start by saying, I'm really worried about this child. I have a very sick child, so we've gotten their attention right away and that's something we're not going to forget now.” S03M, FG1.1*

#### Theme 4: Feeling More Prepared

Students felt that they had received a good foundation in pediatrics. Their perspectives of pediatrics changed. The described feeling less anxious about the upcoming clinical placement and feeling more confident in their ability to communicate in a pediatric setting.

“*...I think all rotations should have these simulated things in the first week just to ease you in and give you an experience of how it would be like to practice...” S31M, FG4.1*

Students also described how they had gained a better understanding of the culture of pediatric medicine and what to expect in clinical practice.

“*Even the culture around pediatrics to know before we go into [Hospital], like having interacted with the tutors, now I'm really looking forward to it. Whereas beforehand I was a little bit apprehensive...” S33F, FG4.2*

This better understanding changed their perspectives toward clinical placement describing that the “*deep end”* was now “*more shallow” (S19M, FG3.1)*. Through experiencing uncertainty yet navigating the experiences successfully helped reassure students that they could also manage challenges on clinical placement. Having had the opportunity to see simulations of an acutely unwell child helped them feel more equipped for the potentially upsetting situations they may encounter in the clinical setting.

### Child Health Literacy Assessment

The results of the child health literacy assessment are from the pre-intervention assessment only. Due to the COVID-19 pandemic it was not possible to administer the assessment post-intervention. All questions on the health literacy assessment had the majority of children responding positively (Figure 2). The question which had the largest positive response was Question 6 (Finding out which food is healthy for you) (95%), followed by Question 10 (Understand what your parents/guardians tell you about your health) (92%). The question which had the largest negative response was Question 7 (Understanding what your doctor says to you) (27%), followed by Question 2 (Knowing what to do to get well quickly when you have a cold) (26%). The question to which the largest number of children responded “Don't Know” was Question 8 (Understanding why you sometimes need to see a doctor even though you are not ill).

## Discussion

We investigated the impact of implementing a novel course that integrated simulation-based learning with interactions with healthy children in advance of clinical placement. We have two main findings: (a) our intervention supported students' learning of pediatric communication skills, and (b) our intervention helped students feel more prepared for their pediatric clinical placement. Our study is one of the first to create an educational intervention to enhance medical students' pediatrics teaching by combining interactions with healthy children outside of the clinical setting with more traditional simulation-based approaches. Similar interventions to support the development of pediatric communication skills in undergraduate medical education involved simulated patient encounters (2, 23) or a community based pediatric health literacy intervention (29), but none of these studies combined the different teaching modalities.

### Pediatrics in the Context of Communication Skills Training

Communication skills teaching is integrated into the medical curriculum in RCSI from year one. Throughout the first 3 years students participate in small group tutorials and simulated patient encounters to learn basic history taking skills in a largely systems-based approach according to the Calgary-Cambridge model (30). Students participate in specialty specific communication skills in fourth year during their clinical rotations. The timing of their pediatric rotation would determine which other specialties students had already completed in fourth year prior to their pediatric rotation, however, for most students their pediatric rotation is the first time they spend significant time interacting with children in a clinical setting. While some of the students' concerns about clinical placement are common to all placements, others differ between specialties, with the triadic nature of communication of particular concern in pediatrics (7).

While for other clinical rotations involving adult patients, simulated patients are an established part of teaching communication skills, the challenges concerning the engagement of children as simulated patients requires a different approach in pediatrics. In our intervention we have shown the impact of combining more traditional simulation-based activities, including simulated patients (as parents) and mannequins as patients, which are common place in adult medicine training, with more novel approaches to experiential learning, specific to pediatrics, involving interactions with healthy children outside the clinical setting. Providing opportunities for medical students to interact with healthy children of different age-groups allowed students to become more aware of the development stages of healthy children, develop the skills needed to communicate with different age groups and become more aware of the concerns of parents. While students reported an improvement in their comfort completing a psychosocial (HEADSS) assessment with an adolescent, they did not have the opportunity to interact with adolescents, only the simulated parent of an adolescent. One possible explanation for this improvement is that many of the students would not have known what a HEADSS assessment was at the start of the week since it is unique to pediatrics. Familiarity with the terminology and tool allowed students to feel more comfortable with it. The intervention could be further enhanced by collaborating with a local post-primary school to provide opportunities for direct interactions with adolescents.

### Curricular Factors

There were a number of elements in the design of the intervention that students specifically identified as helpful for them. While we studied these in the context of pediatric teaching, they are applicable to experiential learning in other specialties.

The clinical setting can be a stressful environment for learning due to the elevated risks when dealing with sick children. When students already have concerns about their own abilities, this additional concern, can negatively impact on learning at the start of placement (5). In our study, the medical students' concerns about their own ability were evident through both the pre-intervention questionnaire and the FGDs. The students gave themselves low ratings on most of the questions on the pre-intervention questionnaire and during the FGDs the students described distinctly their anxiety toward their clinical placement. In our intervention the safe and supportive learning environment afforded opportunities for students to put theory into practice and make mistakes without any major implications either to the welfare of a child or to their overall grades for pediatrics.

Self-reflection through video-review was a transformative experience for many students. They valued the opportunity to separate their learning from the doing. Due to the high cognitive load of a new situation students spoke about having difficulty remembering the scenario afterwards, describing it as “*erased”* from their mind. Students explained that while immersed in the scenario they were not thinking about how it was going, they were just focusing on what they were doing. Having a recording of themselves to watch afterwards allowed them focus on learning at a separate time from doing the activity itself. With reflective observation a key element of experiential learning theory (12), our findings extend our understanding of how students engage in reflective observation and how technology can support and enhance this process.

Often in clinical settings students are paired with just one other student and are not afforded as many opportunities for group learning. In situations where students were less confident they appreciated the opportunity to watch their peers in action before they participated themselves. They also appreciated watching other students to get different ideas of how to approach a situation. While the traditional understanding of experiential learning focused on reflective observation (12), the learning process as described by the students also aligns with vicarious observational learning (13). Their learning occurred through a combination of these processes described by the two theories. While Johnson (14) suggests that learning in simulation by observation is the same as learning by participation, our study indicates that they differ but influence learning positively. This finding has implications for faculty designing interventions: Both doing and watching provide learning experiences for students, but providing one without the other may leave gaps. By providing doing and observing experiences students can relate their abilities to others and gain a broader understanding of how best to approach a situation.

Students valued timely multi-source feedback. The presence of feedback from multiple sources, tutor, peer, SP and their own reflection allowed them to develop insight into their performance but also understand how their perspective compared to others. Students valued having dedicated time to have discussions with tutors to help figure out different ways to approach situations. Feedback from the SP helped students to understand the importance of empathy and transformed the experience from a tick box exercise to an experience where students learned about how a parent might feel in the situation.

Finally, the structure of the teaching influenced students' perceptions of realism. Elements which added realism to the situation allowed students to engage better with the learning. Students valued how convincing the tutors were in embedded participant roles, how realistic the movements and physical signs were on the neonatal mannequin and how well the simulation environment resembled a clinical setting. Aspects of the scenarios that they found unrealistic presented challenges for them. Students found it distracting that no child was present with the simulated parent during the history taking consultation. Even though they knew they were speaking with a parent they had a natural instinct to interact with the child who was not present.

### Preparation for Clinical Placement

Our study confirms previous findings that students can find the transition to clinical placement unpleasant (5). The intervention presented in our study demonstrates how experiential learning prior to clinical placement, can support this transition in pediatrics. The effectiveness of the intervention is evident through the improved student self-reported scores on the pre-post questionnaire and through the insights afforded in the FGDs. In the FGDs students described the various skills, both general and pediatric specific, that they developed which helped them feel more prepared for their clinical placement. They also spoke about gaining greater insight into their own ability, which helped them become more confident. Their increased perceived competence and confidence may help explain why their ratings on the pre-post questionnaire improved for three of the four rotations. For the group in rotation one, the median change in pre post ratings improved for just one question, this question concerned their comfort at completing a psychosocial assessment with an adolescent. Their pediatrics rotation was the first major fourth year clinical rotation for this group so perhaps they could not see how they could translate the learnings outside the clinic into practice. All the other rotations would have seen how, on other clinical placements, they could apply learnings from educational settings to clinical practice. There was one question on the pre-post questionnaire where students reported a decrease in perceived ability. This question concerned their comfort using EBM to motivate patients. In the FGDs, students discussed being worried about managing difficult scenarios with demanding parents or parents who were anti-vaccination. Through the experiences afforded to the students during the week perhaps they gained a greater understanding of the complexity of implementing EBM in practice.

Many students came into their pediatric clerkship with concerns about the challenges that may be presented to them in the pediatric clinical setting. The opportunity to deal with some of these concerns in a safe experiential learning environment in advance of clinical placement helped to alleviate many of the students' concerns. Even students who came to placement with confidence from previous extracurricular experience with children appreciated the opportunity to “ease in” to placement. Having teaching in advance of placement does make the transition less abrupt (4). A lack of knowledge related to specialties contributes to students describing the start of placement as unpleasant (5). Through our intervention students developed skills and a better understanding of the pediatric clinical workplace which helped to alleviate concerns about the transition to clinical placement.

### Community Engaged Learning

Our findings highlight the need for further interventions to help children from DEIS schools to understand doctors. The question to which the largest percentage of children responded negatively, on the health literacy questionnaire, related to understanding what their doctor says to them. We could not measure the change in children's perspective after the intervention due to the restrictions on education at the start of the COVID-19 pandemic, but this aspect would be interesting for future study. Exposure to medicine and healthcare workers in training through community engaged learning (21) has the potential to improve children's understanding of topics relating to healthcare. Firsthand experience in talking to children about health related topics gives medical students valuable insights about how to engage with children, how to use understandable age-appropriate language and how to deal with the unexpected.

### Limitations

Participation in the pre-post questionnaires and the FGDs was voluntary which means that we may have missed some perspectives. This could mean that the results are biased toward participants who were favorable toward the course as those less favorable may not have volunteered. The low response rate for the post-intervention questionnaire was likely because this was completed in the students' own time after the week was complete. At that point, students' attention had shifted to their imminent clinical placement and the arrangements they needed to make in advance of placement. While the post-intervention questionnaire does include participants from each of the four rotations, the results are only representative of those who responded and a higher response rate may have given us perspectives that were more diverse. A more objective measure of student ability before and after the intervention may have given a clearer understanding of baseline ability and change in ability. While the results of our study show how the intervention changed students' perspectives in advance of their clinical placement, capturing students' perspectives after clinical placement may have given greater insights into whether the intervention truly prepared them for clinical placement in retrospect and is an area for potential future work. The children who participated in the workshops and completed the health literacy assessment were from DEIS schools and their responses would not be representative of the general population. A broader assessment of child health literacy may be beneficial. The impact of the COVID-19 pandemic meant that we had to exclude the fifth rotation of students from the study due to major changes to the teaching. Additional student perspectives, especially those completing pediatrics as their final rotation would have added to the study.

## Conclusion

Because of their patient-centered nature, pediatric clerkships represent an irreplaceable learning experience. To address the challenges of workplace learning and variations in students' clinical experience, pediatric educators can supplement clerkships with learning in student-focused environments. This can be achieved by combining simulation with interactions with healthy children to allow students to develop communication skills in a safe and structured manner. Key curricular elements which students found supported their learning were: opportunities to put theory into practice; peer learning; timely multi-source feedback and a safe, supportive environment. The use of technology enhanced reflective observation and afforded students additional opportunities for learning after the experience. Medical students who participated in the intervention reported a greater understanding and confidence related to the skills needed for effective communication in pediatric settings. Community engaged learning has the potential to provide a cost neutral and reciprocal method of improving children's understanding of health related topics, whilst also helping future doctors learn how to effectively communicate with children.

## Data Availability Statement

The raw data supporting the conclusions of this article will be made available by the authors, without undue reservation.

## Ethics Statement

This study, which involved human participants, was reviewed and approved by the RCSI Research Ethics Committee. Written informed consent to participate in this study was provided by the medical students. For the children who participated, written informed consent to participate was provided by the participant's legal guardian/next of kin, as well as written assent from the child.

## Author Contributions

CS, DOL, and MK contributed to the design of the teaching intervention and research study. CS, DOL, MK, and CC contributed to the ethics submission. CS and MK coordinated participant recruitment. CS, CM, and MK coordinated data collection. CS, MA, CC, and WE contributed to data analysis. CS wrote the manuscript with contributions from MA, DOL, CC, CM, and WE. All authors contributed to manuscript revisions and read and approved the submitted version.

## Funding

RCSI University of Medicine and Health Sciences funded this study.

## Conflict of Interest

CS, CC, CM, and WE were affiliated with RCSI SIM. RCSI SIM is a CAE Healthcare Centre of Excellence and receives unrestricted funding from CAE Healthcare to support its educational and research activities. The remaining authors declare that the research was conducted in the absence of any commercial or financial relationships that could be construed as a potential conflict of interest.

## Publisher's Note

All claims expressed in this article are solely those of the authors and do not necessarily represent those of their affiliated organizations, or those of the publisher, the editors and the reviewers. Any product that may be evaluated in this article, or claim that may be made by its manufacturer, is not guaranteed or endorsed by the publisher.
