# Supplementary material for: Preparing for Pediatrics: Experiential Learning Helps Medical Students Prepare for Their Clinical Placement
Source: Front Pediatr. 2022 Mar 4;10:834825. doi: 10.3389/fped.2022.834825 (PMC8931532; doi:10.3389/fped.2022.834825)
Supplement: Supplementary file 1 [file Table_1.DOCX]

**Appendix A**

**List of Scenarios**

| **Simulated Parent Scenarios** | **Team-Based Simulation Scenarios** |
| --- | --- |
| Consultation with a parent of a 3 year old child with asthma | Care of a 5 week old infant with sepsis |
| Consultation with a parent of a 13 year old child with diabetes | Care of a 6 month old infant with bronchiolitis |
| Consultation with a parent of 3 year old child with transient synovitis | Care of a 2 day old infant with jitteriness due to maternal substance use |
| Consultation with a parent of a 2 year old child with febrile seizures | Care of a 7 week old pre-term infant (33 weeks) with abdominal obstruction |
| Consultation with a parent of a 20 month old child with isolated gross motor delay |  |
