# Supplementary material for: Preparing for Pediatrics: Experiential Learning Helps Medical Students Prepare for Their Clinical Placement
Source: Front Pediatr. 2022 Mar 4;10:834825. doi: 10.3389/fped.2022.834825 (PMC8931532; doi:10.3389/fped.2022.834825)
Supplement: Supplementary file 2 [file Table_2.DOCX]

**Appendix B**

**Simulated Patient (SP) Training Overview**

| **RCSI Simulated Patient Training Programme** | |
| --- | --- |
| Session 1 | Professionalism  Concept of simulation  Simulation in healthcare education  Introduction to cases |
| Session 2 | Healthcare consultations and curriculum overview  Attributes of a good SP  SP Case Development – The backstory  Communication skills |
| Session 3 | Hybrid simulation  Feedback  Confidentiality  Calgary Cambridge model  Case practice |
| Session 4 | SP performance assessment  How to portray emotions  Review |
