# Supplementary material for: Preparing for Pediatrics: Experiential Learning Helps Medical Students Prepare for Their Clinical Placement
Source: Front Pediatr. 2022 Mar 4;10:834825. doi: 10.3389/fped.2022.834825 (PMC8931532; doi:10.3389/fped.2022.834825)
Supplement: Supplementary file 3 [file Table_3.DOCX]

**Appendix C**

**Subset of Questions from the Paediatric Consultation Skills Assessment Tool (PCAT)** (Howells *et al.* 2010)

The questions were a subset of the PCAT and were selected to align with what the medical students were asked to complete during the consultation. Responses were on a seven point descriptive scale, refer to marking key in Howells *et al.* 2010.

**Building the Relationship**

Q1: Non-verbal skills: eye contact, open posture, avoids writing / reading

Q2: Is empathetic and supportive shows concern, responds to family's predicament

**Initiating the session**

Q3: Introduces self, clarifies role, determines who is present

Q4: Identifies reasons for the consultation - the doctor's and family's

Q5: Screens for other problems and negotiates the agenda for the consultation

**Gathering Information**

Q6: Listens attentively, facilitating verbally and non-verbally

Q7: Picks up and responds to verbal and non-verbal cues

Q8: Uses appropriate questioning techniques (e.g. open / closed questions)

Q9: Explores parent / child's ideas, concerns, feelings, expectations

**Explanation and Planning**

Q10: Tailors amount and type of information for parent/s and child

Q11: Uses skills which aid recall and understanding

Q12: Incorporates parent / child's perspective into explanation

**Closure**

Q13: Establishes and clarifies next steps with parent/s and child

Q14: Makes contingency plans

**Structuring the interview**

Q15: Uses skills which provide structure (e.g. summarising and signposting)
