# Supplementary material for: Preparing for Pediatrics: Experiential Learning Helps Medical Students Prepare for Their Clinical Placement
Source: Front Pediatr. 2022 Mar 4;10:834825. doi: 10.3389/fped.2022.834825 (PMC8931532; doi:10.3389/fped.2022.834825)
Supplement: Supplementary file 4 [file Table_4.DOCX]

**Appendix D**

**Pre-Intervention and Post-Intervention Questionnaire** (Whitt et al 2014)

Q1. Please rate your comfort level in communicating with parents and family members of young children.

         1 2 3 4 5 6 7 8 9 10

Not comfortable                   Very comfortable

Q2. Please rate how effective you are at communicating with parents and family members of young children.

         1 2 3 4 5 6 7 8 9 10

Not effective                     Very effective

Q3. Please rate your comfort level in managing a confrontational situation involving family members with differing opinions.

         1 2 3 4 5 6 7 8 9 10

Not comfortable                   Very comfortable

Q4. Please rate how effective you are at managing a confrontational situation involving family members with differing opinions.

         1 2 3 4 5 6 7 8 9 10

Not effective                     Very effective

Q5. Please rate your comfort level in completing a psychosocial (HEADSS) assessment with an adolescent.

         1 2 3 4 5 6 7 8 9 10

Not comfortable                   Very comfortable

Q6. Please rate how effective you are at completing a psychosocial (HEADSS) assessment with an adolescent.

         1 2 3 4 5 6 7 8 9 10

Not effective                     Very effective

Q7. Please rate how comfortable you are at using Evidence-Based Medicine when motivating patients.

         1 2 3 4 5 6 7 8 9 10

Not comfortable                   Very comfortable

Q8. Please rate how effective you are at using Evidence-Based Medicine when motivating patients.

         1 2 3 4 5 6 7 8 9 10

Not effective                     Very effective
