# Supplementary material for: Preparing for Pediatrics: Experiential Learning Helps Medical Students Prepare for Their Clinical Placement
Source: Front Pediatr. 2022 Mar 4;10:834825. doi: 10.3389/fped.2022.834825 (PMC8931532; doi:10.3389/fped.2022.834825)
Supplement: Supplementary file 5 [file Table_5.DOCX]

**Appendix E**

**Topic Guide - Outline of Focus Group Discussions**

1. Their previous thoughts and experiences regarding communication in pediatric settings

2. The challenges they have faced during the week of experiential learning

3. The lessons they felt they learned from the week

4. The aspects of the week they found most useful

(Relate each of the above to the various elements of the week)

(Prompts regarding confidence, language, engagement, teamwork, leadership)
